# Tobacco Industry Manipulation of Tobacco Excise and Tobacco Advertising Policies in the Czech Republic: An Analysis of Tobacco Industry Documents

**DOI:** 10.1371/journal.pmed.1001248

**Published:** 2012-06-26

**Authors:** Risako Shirane, Katherine Smith, Hana Ross, Karin E. Silver, Simon Williams, Anna Gilmore

**Affiliations:** 1 Department for Health, University of Bath, Bath, United Kingdom; 2 Global Public Health Unit, School of Social & Political Science, University of Edinburgh, Edinburgh, United Kingdom; 3 International Tobacco Control Research, American Cancer Society, Atlanta, Georgia, United States of America; 4 Department of Social and Policy Sciences, University of Bath, Bath, United Kingdom; San Diego State University, United States of America

## Abstract

**Background:**

The Czech Republic has one of the poorest tobacco control records in Europe. This paper examines transnational tobacco companies' (TTCs') efforts to influence policy there, paying particular attention to excise policies, as high taxes are one of the most effective means of reducing tobacco consumption, and tax structures are an important aspect of TTC competitiveness.

**Methods and Findings:**

TTC documents dating from 1989 to 2004/5 were retrieved from the Legacy Tobacco Documents Library website, analysed using a socio-historical approach, and triangulated with key informant interviews and secondary data. The documents demonstrate significant industry influence over tobacco control policy. Philip Morris (PM) ignored, overturned, and weakened various attempts to restrict tobacco advertising, promoting voluntary approaches as an alternative to binding legislation. PM and British American Tobacco (BAT) lobbied separately on tobacco tax *structures,* each seeking to implement the structure that benefitted its own brand portfolio over that of its competitors, and enjoying success in turn. On excise *levels*, the different companies took a far more collaborative approach, seeking to keep tobacco taxes low and specifically to prevent any large tax increases. Collective lobbying, using a variety of arguments, was successful in delaying the tax increases required via European Union accession. Contrary to industry arguments, data show that cigarettes became more affordable post-accession and that TTCs have taken advantage of low excise duties by raising prices. Interview data suggest that TTCs enjoy high-level political support and continue to actively attempt to influence policy.

**Conclusion:**

There is clear evidence of past and ongoing TTC influence over tobacco advertising and excise policy. We conclude that this helps explain the country's weak tobacco control record. The findings suggest there is significant scope for tobacco tax increases in the Czech Republic and that large (rather than small, incremental) increases are most effective in reducing smoking.

*Please see later in the article for the Editors' Summary*

## Abstract 2

Risako Shirane and colleagues examined the the Legacy Tobacco Documents Library and found evidence of transnational tobacco company influence over tobacco advertising and excise policy in the Czech Republic, a country with one of the poorest tobacco control records in Europe.

## Editors' Summary

**Background:**

Every year, about 5 million people die from tobacco-related diseases and, if current trends continue, annual tobacco-related deaths will increase to 10 million by 2030. Faced with this global tobacco epidemic, national and international bodies have drawn up conventions and directives designed to control tobacco. For example, European Union (EU) Directives on tobacco control call for member states to ban tobacco advertising, promotion, and sponsorship and to adopt taxation policies (for example, high levels of tobacco excise tax) aimed at reducing tobacco consumption. Within the EU, implementation of tobacco control policies varies widely but the Czech Republic, which was formed in 1993 when Czechoslovakia split following the 1989 collapse of communism, has a particularly poor record. The Czech Republic, which joined the EU in 2004, is the only EU Member State not to have ratified the World Health Organization's Framework Convention on Tobacco Control, which entered into force in 2005, and its tobacco control policies were the fourth least effective in Europe in 2010.

**Why Was This Study Done?:**

During the communist era, state-run tobacco monopolies controlled the supply of cigarettes and other tobacco products in Czechoslovakia. Privatization of these monopolies began in 1991 and several transnational tobacco companies (TTCs)—in particular, Philip Morris and British American Tobacco—entered the tobacco market in what was to become the Czech Republic. In this socio-historical study, which aims to improve understanding of both effective tobacco excise policy and the ways in which TTCs seek to influence policy in emerging markets, the researchers analyze publically available internal TTC documents and interview key informants to examine efforts made by TTCs to influence tobacco advertising and tobacco excise tax policies in the Czech Republic. A socio-historical study examines the interactions between individuals and groups in a historical context.

**What Did the Researchers Do and Find?:**

The researchers analyzed 511 documents (dated 1989 onwards) in the Legacy Tobacco Documents Library website (a collection of internal tobacco industry documents released through US litigation cases) that mentioned tobacco control policies in the Czech Republic. They also analyzed information obtained from sources such as tobacco industry journals and data obtained in 2010 in interviews with key Czech informants (including a tobacco industry representative and a politician). The researchers' analysis of the industry documents indicates that Philip Morris ignored, overturned, and weakened attempts to restrict tobacco advertising and promoted voluntary approaches as an alternative to binding legislation. Importantly, while the internal documents show that Philip Morris lobbied for a specific excise tax (a fixed amount of tax per cigarette, a tax structure that favors the expensive brands that Philip Morris mainly markets), the European strategy employed at that time by British American Tobacco was to lobby for a mixed excise structure that combined an “ad valorem” tax (a tax levied as a proportion of price) and a specific tax, an approach that favors a mixed portfolio of tobacco brands. By contrast, the documents show that TTCs collaborated in trying to keep tobacco taxes low and in trying to prevent any large tax increases. This collective lobbying successfully delayed the tobacco tax increases required as a condition of the Czech Republic's accession to the EU. Finally, the interview data suggest that TTCs had high-level political support in the Czech Republic and continue actively to attempt to influence policy.

**What Do These Findings Mean?:**

These findings provide clear evidence that Philip Morris and British American Tobacco (the two TTCs that have dominated the Czech market since privatization of the tobacco industry) have significantly influenced tobacco advertising and excise policy in the Czech Republic since 1989. The findings, which also suggest that this influence is ongoing, help to explain the Czech Republic's poor tobacco control record, which was reflected in a fall in the real price of cigarettes between 1990 and 2000. More generally, this study provides valuable insight into how TTCs might try to influence policy in other emerging markets. Improvements in global tobacco control, the researchers conclude, will be possible only if efforts are made to protect tobacco control policies from the vested interests of the tobacco industry, a principle enshrined in the WHO Framework Convention on Tobacco control, and if public and political attitudes to the industry shift.

**Additional Information:**

Please access these Web sites via the online version of this summary at http://dx.doi.org/10.1371/journal.pmed.1001248.

- The World Health Organization provides information about the dangers of tobacco (in several languages) and about its Framework Convention on Tobacco Control
- For information about the tobacco industry's influence on policy, see the 2009 World Health Organization report Tobacco interference with tobacco control
- The Framework Convention Alliance more information about the FCTC
- Details of European Union legislation on excise duty applied to manufactured tobacco and on the manufacture, presentation and sale of tobacco products are available (in several languages)
- The Legacy Tobacco Documents Library is a searchable public database of tobacco company internal documents detailing their advertising, manufacturing, marketing, sales, and scientific activities
- The UK Centre for Tobacco Control Studies is a network of UK universities that undertakes original research, policy development, advocacy, and teaching and training in the field of tobacco control
- SmokeFree, a website provided by the UK National Health Service, offers advice on quitting smoking and includes personal stories from people who have stopped smoking
- Smokefree.gov, from the US National Cancer Institute, offers online tools and resources to help people quit smoking and not start again

## Introduction

The collapse of communism in 1989 prompted the split of Czechoslovakia into the Czech Republic and Slovakia in 1993 [1]. It also prompted economic reforms that led to the privatisation of the state-run tobacco monopolies, Tabak Akciová Společnost (hereinafter referred to as “Tabak”) in the Czech Republic and Československý Tabakový Priemysel in Slovakia. This provided opportunities for transnational tobacco companies (TTCs), which are known to have exploited privatisation processes elsewhere [2],[3],[4],[5].

More recently, in 2004, the Czech Republic joined the European Union (EU), which brought with it a requirement to implement EU Directives on tobacco control [6]. Despite this, tobacco control remains weak in the Czech Republic. Between 1990 and 2000, real cigarette prices fell [7] and in 2010, its tobacco control policies were ranked the fourth least effective in Europe [8]. Furthermore, senior political figures publically support the tobacco industry. For example, while opening Philip Morris' (PM) Kutna Hora factory in September 2010, the Czech President Vaclav Klaus commended PM's contribution to the country and challenged EU tobacco regulations, reportedly stating: “I support the fight against restrictions on smoking. […] This is stupid; it is unreasonable and something that politicians should not do” [9]. The Czech Republic also remains the only EU Member State that has not yet ratified the Framework Convention on Tobacco Control (FCTC). Despite this worrying state of tobacco control, no previous studies have examined tobacco industry influence in the Czech Republic.

This paper addresses this gap. Examining the period from 1989 onwards (thus covering the key political events of privatisation and EU accession), it aims to explore three main issues: (i) the tactics of market entry; (ii) TTC influence on tobacco marketing restrictions; and (iii) TTC influence on tobacco taxation in the Czech Republic. In so doing, it aims to improve understanding of both effective excise policy and the ways in which TTCs seek to influence policy in emerging markets. Taxation is of particular interest because it is highly effective in reducing tobacco consumption [10],[11],[12],[13] and raises revenue for governments [14],[15]. It also influences TTCs' competitiveness, because different excise systems favour different brand portfolios (the collection of brands that each company sells, usually across a range of price segments; see Table 1). Nevertheless, only a limited literature explores TTCs' efforts to influence tax policies, and most studies focus on North America and deal exclusively with tax levels [16].

**Table 1:** Tobacco excise structures and their effects on tobacco companies.

Excise structure	Characteristics	Effects on companies
Specific	• Levied as a fixed amount (per cigarette weight/pieces/pack/carton)**Advantages**• Ease of implementation• Likely to ensure stable and predictable tax revenue• Reduces the gap between cheap and expensive brands and thus the motivation for down-trading• Compared to ad valorem taxes, tends to encourage overshifting of tax as any increase in price (over the tax increase) will be accrued in profit• Specific tax increases tend to reduce consumption more than equivalent ad valorem tax increases**Disadvantages**• Not automatically indexed for inflation and therefore needs regular rate-adjustment• If levied per pack/stick, can encourage TTCs to produce longer, King-Size cigarettes without additional tax burden.	• TTCs selling expensive brands favour this structure because, as a % of price, the burden on expensive cigarettes is lower than on cheaper cigarettes & the price gap is narrowed. This encourages smokers to smoke more expensive brands.• TTCs can raise the base cigarette price (and thus their profit margin) without a corresponding tax increase, as tax levels are independent of retail price. Thus specific prices enable greater profitability, certainly in established markets.
Ad-valorem	• Levied as a percentage of price**Advantages**• Tax amount automatically indexed for inflation if the industry raises its base price in line with inflation.• An increase in profit margin or in the costs of inputs automatically increases the amount of tax paid by consumers.**Disadvantages**• Tax revenues may not be as predictable and stable as specific taxes (TTCs can adjust their cigarette prices to minimise their tax liability).• Increases the price gap between cheap and expensive products, which encourages product substitution after a price/tax increase thus diminishing the impact of the tax increase.• May encourage companies with expensive brands to reduce their prices in order to minimise the price gap.	• TTCs with cheaper brands usually favour this structure, as it can lead to a large price discrepancy between their cheap brands and other, more expensive brands.
Mixed	• Incorporates specific and ad valorem components**Advantages and disadvantages**• Mixed systems incorporate some of the advantages but also some of the disadvantages of specific and ad valorem systems. The extent to which they do so depends on whether they are weighted towards specific or ad valorem elements.	• TTCs with brands of mid or broad price-range usually prefer a mixed structure.
Tiered	• Tobacco excise may also be *tiered*, with different tax levels applied to different categories of tobacco product (categorised by, for example, cigarette length, source of production, whether filtered or unfiltered).	• TTCs with brands that fall into the lower tax tiers usually favour this structure, as it discriminates in their favour.

## Methods

This study used a qualitative design, which centred on analysing internal tobacco industry documents released through a series of litigation cases in the US. These data were supported by, and triangulated with, an analysis of other publicly available documents and nine interviews with key informants. The tobacco industry documents were searched via the Legacy Tobacco Documents Library website (http://legacy.library.ucsf.edu/). The date range was restricted to 1989 onwards (covering privatisation and accession to the EU), although undated documents were also included. A broad initial search using the following string: Czech* AND (tax OR taxation OR excise OR “ad valorem”) yielded 4,785 documents, all of which were briefly examined. The surrounding Bates numbers of particularly relevant documents were also checked. Relevant documents were downloaded to an EndNote library and analysed in chronological order, after which further searches were undertaken to follow up specific events, organisations, and individuals. As the initial analysis indicated that TTCs had also been involved in influencing non-tax policies, notably tobacco advertising bans, additional searches also focused on these activities. 511 documents were analysed in detail, using the tobacco document methodology developed by Gilmore [17], which is informed by a socio-historical approach [18],[19]. The majority of documents identified as relevant to this work were PM and British American Tobacco (BAT) documents. This reflects both the nature of the document collections (access to TTC documents is limited mainly to those of PM and BAT, as documents of companies that were not party to the US litigation are not publically available) and the fact that these two companies have dominated the Czech market since transition. The most recent, relevant document identified was dated 2004/5, although only 32 documents date from 2000 onwards.

Additional data sources, including tobacco industry journals, newspaper articles (obtained via Nexis database searches), and market reports (e.g. Euromonitor, ERC Statistics) were used to triangulate this analysis and to provide more recent data. Semi-structured, key informant interviews were undertaken in November 2010. Interviews were conducted face-to-face in Czech by a native Czech speaker with tobacco control expertise. Potential interviewees were selected by a snowball technique; eight out of 11 individuals approached agreed to be interviewed and for their interview to be recorded. Attempts were made to include all relevant stakeholder groups, and we were successful in interviewing a broad spectrum including a public health expert, a civil servant, a tobacco industry representative, a politician, and a political advisor. The primary topics in the interview schedule included changes to and influences on excise policy at EU and national levels including individual and collective efforts of the TTCs to influence policy, dealings with the tobacco industry or third parties representing the industry, provision of advice on tobacco control policy, tobacco smuggling, industry pricing strategies, EU accession negotiations and influence thereon, and the FCTC. Interview transcripts were analysed using the Framework approach [20],[21], in which qualitative data are coded and organised according to themes and sub-themes, allowing for the incorporation of both a priori themes and those which emerge through the analytical process. Observations from interview notes were used to help provide a context for the analysis.

## Results

### TTCs Entry to the Czech Market and Efforts to Achieve Market Dominance

In 1991, the government commenced a process of privatising the Czech and Slovak tobacco monopolies. Multiple TTCs (PM, BAT, Reemtsma, Rothmans, and R.J. Reynolds [RJR]) prepared to enter the market [22],[23], which was deemed of strategic importance because of its central European location, favourable economic prospects, and borders with other former socialist countries the TTCs hoped to access [24],[25],[26]. Both PM and BAT perceived shaping the tobacco tax system as an important step towards securing market share and future profits [27],[28],[29],[30].

Prior to market opening, legal TTC sales in Czechoslovakia occurred only through hard currency and Duty Free Shops [31], which were unavailable to the general population. However, by 1991, documents suggest that cigarette smuggling into Czechoslovakia was commonplace; a BAT agent estimated that 70% of BAT's and 90% of PM's cigarettes on the market were “smuggled” [32]. Moreover, tobacco marketing was already widespread with TTC brands among those heavily marketed [32].

In March 1992, in the western part of Czechoslovakia (now the Czech Republic), Tabak was put out to tender [22],[33]. PM, which had a close working relationship with the Czechoslovakian government and Tabak (through a licensing agreement established in 1987 to produce PM's most prominent brand, Marlboro [34],[35]), successfully convinced the government to abandon its plan to break up Tabak [22],[36],[37]. In April 1992, PM acquired a 30% share (the largest US investment in the Czech history at the time) [35] and by 1993, it had gained a majority holding (67.4%) [27] and thus monopoly power over the domestic market.

The Czech government had planned to abolish the law giving PM (via ownership of Tabak) a monopoly on cigarette production [38] but, according to BAT, PM attempted to maintain its market dominance by trying to impede this change [36],[39],[40]. BAT repeatedly lobbied key government officials [39],[41],[42],[43],[44], arguing that failure to change the legislation would “inhibit the establishment” [40] of the “truly free market for tobacco products” [40] to which the Czech government had committed [40]. Documents suggest that BAT's lobbying efforts (on which it spent at least £120,000 by June 1993 [45]) were successful [45],[46],[47],[48],[49], ultimately enabling BAT to establish a small production facility in May 1995 [50],[51].

PM therefore turned its attention to maintaining its market leadership via other means [52]. A notable example is PM's support for and direct engagement with the Czech authorities to introduce a tax stamp system in the mid-1990s [53],[54],[55],[56], demonstrating its apparent concern about “competition from illegally imported cigarettes” [57], which included competitor TTC brands [58]. To PM's satisfaction [54], the tax stamp system was implemented in 1994 and helped contain levels of contraband at around 3% of sales for a few years [54],[59].

### TTC Efforts to Influence Marketing Restrictions

Prior to market entry, some marketing restrictions were introduced in Czechoslovakia, notably a 1992 Consumer Protection Act, which stated that it was “forbidden to advertise tobacco products” [60]. However, TTCs ignored this, posting large adverts on billboards, store fronts, and city trams [61],[62]. PM claimed that the Act could not be enforced until officials had further defined it [62] and pursued “all available means to obtain a favourable amendment” [63],[64],[65],[66],[67]. It used a previously established organisation, Libertad, which, although fully funded by PM, positioned itself as not-for-profit [68],[69],[70]. Supported by the global public relations company Burson-Marsteller [70], Libertad helped frame freedom to advertise tobacco products as a matter of commercial free speech [64],[71],[72]. The campaign was successful and the advertising ban was formally cancelled in July 1993 [48],[63]. PM subsequently worked to produce a voluntary code of conduct [48],[68],[73], presumably to decrease the likelihood that another legislative ban would be proposed (a tactic used elsewhere [74]).

However, to PM's apparent surprise, a further advertising ban was passed in December 1993, which PM again worked “to reverse” [53],[75],[76],[77], promoting self-regulation as an alternative [77]. In February 1994, a vote on relaxing the ban was passed, allowing existing tobacco advertising contracts to run until December 1994 or until a new law was passed [78],[79], meaning that, although tobacco advertising was technically banned, it still existed throughout the country [61]. Just days prior to this vote, PM had taken five Czech Members of Parliament (MPs) to a two-day all-expenses paid “briefing trip” to Switzerland [77],[80], where a tobacco and alcohol advertising ban had recently been rejected in a referendum vote following a PM campaign [81]. Two of these parliamentarians voted in favour of amending the ban and the others abstained or were absent [80]. Soon after the vote, the Czech Prime Minister committed the government to completely cancelling the advertising ban and seeking alternative legislation [79].

The government started working on a new advertising law in March 1994, and by April 1994, PM had become directly involved with its development [77]. The new law was approved in October and in line with PM's objective, relied on self-regulation [77]. PM managers regarded this as a success [77],[82] and planned to use a similar, voluntary code to try to overturn the advertising ban still in place in Slovakia [82]. PM documents note that a “behind the scenes approach” helped them achieve success [77]. A key component of this approach was the establishment of the Council for Advertising, an organisation made up of advertisers and the media which was charged with administering an industry marketing code, closely modelled on PM's own internal code [77],[83]. At least two documents suggest PM was involved in establishing the Council for Advertising [77],[83] and another suggests PM helped fund it [84].

By 1994–1995, the Czech Parliament approved an amendment to the Law of Prevention of Alcoholism and Other Drug Addictions, which included a ban on day-time TV and radio advertising for tobacco products [85]. However, the law was rejected by President Havel [86],[87],[88], following “several weeks of intensive lobbying by the industry” [88].

### TTC Efforts to Influence Tobacco Excise Policy

#### Influence on excise policy during privatisation

Both PM and BAT tried to influence tax policy before, during, and after the privatisation process and both generally wanted to minimise tobacco tax *levels* [89],[90],[91],[92]. However, each had different approaches to excise tax *structure*, in line with their contrasting brand portfolios [93],[94],[95],[96]. PM's portfolio is dominated by the premium brand Marlboro. As fully specific excise structures benefit expensive brands (Table 1), PM's objective was to replace the fully ad valorem (proportional) structure then in place (1989–1990: see Table 2) [97] with an entirely specific excise tax structure [93]. Although documents do not specify that a single-rate (i.e., not tiered) specific system was the ultimate objective, we assume this was the case from the objectives PM outline; the specific system was intended to reduce the price gap between Marlboro and others' cheaper brands, in order to eliminate the cheaper brands' “price advantage” [93] and enable consumers “to choose their cigarettes based on product quality and brand image rather than price” (i.e. to choose its more expensive brands) [98]. Accordingly, PM promoted a fully specific excise tax structure after its acquisition of Tabak in 1992 [89].

**Table 2:** Cigarette excise structures and levels in Czechoslovakia/Czech Republic, 1989–2009.

Period (Month/Year)	Ad valorem tax(% of TIRSP^1^)	Specific tax(CK^2^ per 1000)	VAT^3^(%TIRSP)	MinimumTax level(CK per 1000)
1989	71% of turnover^4^	n/a	n/a	n/a
1990	No filter: 67% turnoverWith filter: 75% turnoverSparta (the most popular brand): 84% turnover	n/a	n/a	n/a
1991	n/a	3 tiers based on length & origin<70 mm: 270>70 mm: 460Import: 830	n/a	n/a
1992	n/a	3 tiers based on length & origin<70 mm: 340>70 mm: 575Import: 1040	n/a	n/a
Jan/1993	n/a	2 tiers based on length<70 mm: 270>70 mm: 460	23	n/a
Aug/1993	n/a	<70 mm: 360>70 mm: 460	23	n/a
1994	n/a	<70 mm: 400>70 mm: 500	23	n/a
1995	n/a	<70 mm: 400>70 mm: 500	22	n/a
1996	n/a	<70 mm: 550>70 mm: 650	22	n/a
1997	n/a	<70 mm: 550>70 mm: 650	22	n/a
1998	n/a	<70 mm: 640>70 mm: 740	22	n/a
Jan/1999	n/a	<70 mm: 640>70 mm: 740	22	n/a
July/1999–July/2001	n/a	<70 mm: 670>70 mm: 790	22	n/a
Aug/2001–Dec/2003	22	360	22	790
Jan/2004	23	480	22	<70 mm: 900>70 mm: 960
May/2004^5^	23	480	19	940
July/2005	24	600	19	1130
April/2006	25	730	19	1360
March/2007	27	880	19	1640
Jan/2008	28	1030	19	1920
Feb/2010	28	1070	20	2010

Source: [97],[162],[163],[164],[165],[166],[167],[168],[204],[205],[206],[207],[208].

1 TIRSP stands for Retail Selling Price, all Taxes included.

2 CK stands for Czech Koruna, the official currency of the Czech Republic.

3 VAT stands for Value Added Tax.

4 ‘turnover’ is equivalent to wholesale price.

5 The length of cigarettes is no longer relevant after this date (NB length based taxation was in place to 2001 (on an ad valorem basis), were removed between August 2001 and December 2003, but in early 2004 were reapplied on a specific basis).

BAT, which had a more diverse cigarette price portfolio with a greater emphasis on the economy price segment than PM, wanted to shape the excise tax structure differently [95],[96]. We have been unable to find any documents that clarify precisely what BAT envisioned, but the company's broad European strategy at this time was to achieve a mixed excise structure (combining an ad valorem tax with a significant specific component) [99],[100]. Given the rationale for this system was that it would disadvantage PM's more expensive brands [101], it is likely that BAT was trying to achieve a mixed excise tax structure in the Czech Republic. By 1990, both companies were actively lobbying to influence excise tax policy [25],[102]. In 1991, BAT met with Mr. Antonin Kalina of the Czechoslovakian Ministry of Finance to offer the company's “worldwide tax experience” [103]. However, BAT's lobbying efforts were initially unsuccessful and a specific excise tax structure was implemented in January 1991 (Table 2) [26], which continued to disadvantage its brands [104].

Although the excise structure implemented in 1991 was specific (in line with PM's preferences), it also incorporated three tiers, based on both geographical origin and length of cigarettes (Table 2) [26]. This did not offer PM the advantages of a normal, single-rate specific system (Table 1), given that both long cigarettes (largely produced by TTCs) and imports (on which the TTCs then relied [28],[36]) were taxed most highly. In January 1991, PM became concerned with the discriminatory effects of the tiered structure on foreign brands, which “incur approximately double the tax burden that is applied to domestic filter cigarettes” [28] and claimed it contravened the principles of General Agreement on Tariffs and Trade because it discriminated against foreign goods [28], a tactic PM had previously employed in Hungary [28],[105].

Although the tiered tax structure remained in place until 2001, once PM had acquired Tabak, thereby starting to produce shorter (lower taxed), local cigarette brands, the structure became “extremely beneficial to Philip Morris” [106]. Nevertheless, PM remained unhappy with the significant price gap between Marlboro and other brands [67], as it hindered its broader plan for new markets: to buy up local brands with the long-term goal of trading “consumers up to premium brands,” notably Marlboro [107]. This would increase returns given the greater profit margins generally enjoyed on premium brands. A predominantly specific tax structure and a willingness to temporarily absorb tax increases (to make Marlboro more affordable) were central to this strategy:

“In expansion areas (excluding Japan) affordability is the key issue and managing the price gap between Marlboro and the next pricing category is the critical strategy. To do so requires selective pricing including choosing to absorb tax increases partially or in full.” [107]

Accordingly, PM lobbied the Ministries of Economic Competition and Finance and relevant parliamentary committees [48],[63] with apparent success. PM documents from 1993 and 1994 report that the company obtained a reduction in the tax difference between tiers by raising the tax burden on short cigarettes (Table 2) [63],[67].

In arguing for its preferred excise tax system, PM also tried to “resist any linkage by governments” [108] of health objectives to tobacco taxes, probably because this linkage is recognised by TTCs as providing a rationale for governments to increase tax and/or “earmark” revenue for health programmes [16].

#### Outcomes of TTC influence on excise during privatisation

**Figure 1:**
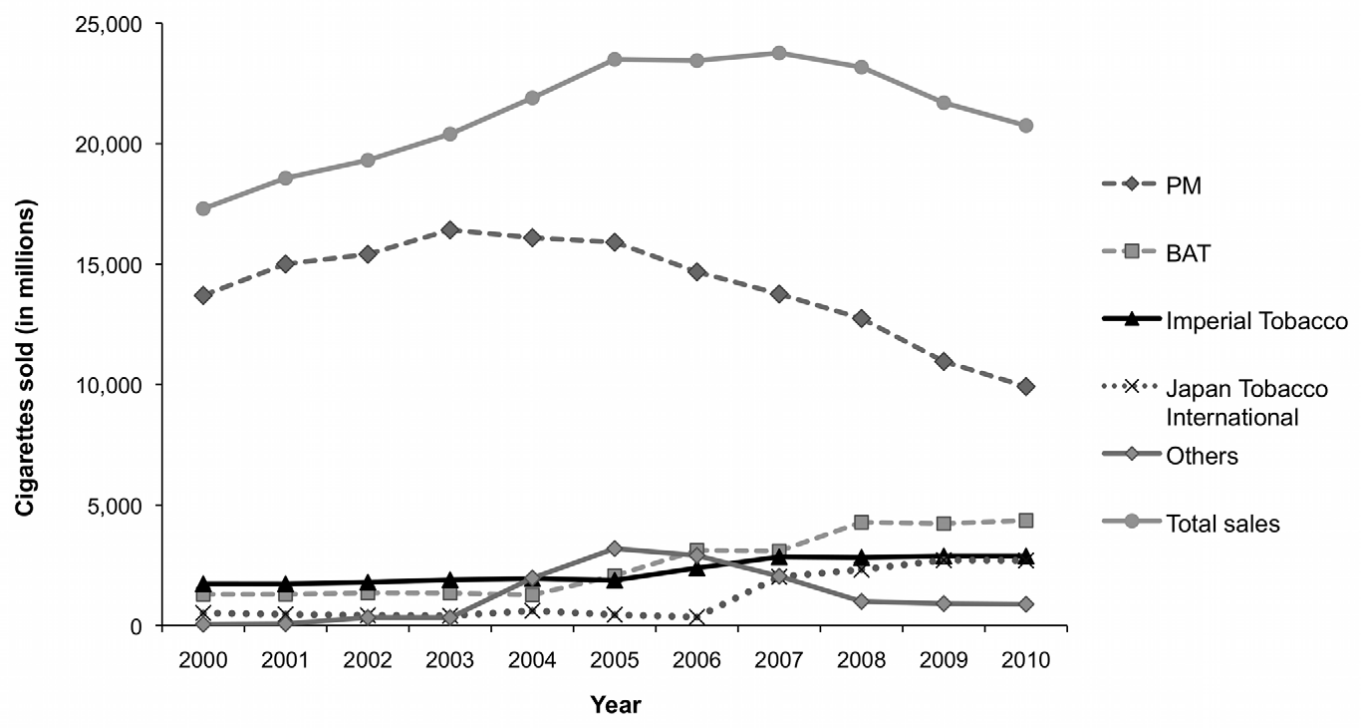
Cigarette sales by volume (in millions) overall and by company in the Czech Republic, 2000–2010. [169]

Although PM did not achieve its ultimate aim of a single-rate specific excise structure, it still successfully influenced excise policy in its favour during the early to mid 1990s, most notably by increasing the tax on short cigarettes and thereby narrowing the difference between the tiers. According to market reports, this appears to have had a direct impact on sales and market shares. The sale of short cigarettes fell rapidly in the mid-1990s, with king-size (i.e. larger, generally international cigarettes [109],[110]) coming to hold over three-quarters of the market [59]. By 2000, PM had achieved an 80% market share (Figure 1) [59].

### Influence on Excise Policy during EU Accession

EU accession required the Czech Republic to implement all EU legislation including EU tobacco tax directives [6]. As such, it was required to implement the EU's minimum excise requirement (a 57% excise tax rate on the Most Popular Price Category [111]) and to put a mixed excise system in place. In the Czech Republic, this required increasing tobacco taxation levels and adding an ad valorem element to the existing specific excise tax. Efforts to influence excise policy during the accession process focused on these two issues.

#### Delaying implementation of the EU minimum excise requirement

In late 1993, aware of the requirement for EU accession states to implement EU tobacco tax directives, the TTCs operating in these countries attempted to overcome their rivalries and develop a united position on tax issues [112],[113]. They recognised that “lack of industry coordination in communicating with relevant officials” [113] on tax issues had previously undermined the industry's credibility [112],[113], and could induce governments to “act against the long-term interests of… the industry” [92]. BAT, which was generally in favour of mixed excise systems (see above), was particularly forthright about the benefits of a united approach, claiming this was “the most effective way to improve industry and BAT ['s] control” in the acceding countries [114]. TTCs began discussing tax issues at Eastern Europe Working Group meetings around 1993 [92],[115],[116] and subsequently established the Central Europe Tax Task Force [113] to promote a “consistent approach and argumentation” [113],[117]. In 1996, the Central Europe Tax Task Force began working to achieve united tax harmonisation goals in Central European countries, including the Czech Republic [113],[117],[118].

One important, unanimously agreed goal of the Central Europe Tax Task Force was to oppose any large-scale increases in total tax incidence [117],[118],[119]. Accordingly, the TTCs aimed to obtain a 5-year derogation period for the Czech Republic's requirement to implement the 57% minimum excise requirement [114],[119], expressing concerns about “unsustainable price increases” [120] and heightened risks of smuggling [120]. A note of a Working Group meeting in February 1996 indicates that members planned to suggest to governments of the Central and East European countries that the 57% minimum requirement might be reduced [120], although we could find no evidence to support this claim. In fact, the history of the development of EU tax directives shows that excise levels have consistently increased, rather than fallen, over time [121]. Later in 1996, all members of the Central Europe Tax Task Force agreed to avoid endorsing, or even mentioning, the 57% minimum requirement when lobbying (see Table 3 for a more detailed overview of the arguments TTCs planned to use to promote the need for derogation) [118],[119]. The industry's desire to avoid any major increases in tax (and thus price) is consistent with financial analyst reports, which indicate that small, gradual tax increases can actually benefit the industry [122],[123]. Analysts suggest this is because, unlike large tax increases, incremental increases have relatively little impact on consumption and can also enable overshifting (where TTCs increase prices on top of the excise increases, thus increasing profits) [124]. TTCs' strong preference for gradual tax increases was acknowledged in two separate interviews:

“They [the TTCs] did not mind gradual increases [during EU accession], but they feared major jumps. They said: if you want an increase by, say, 20%, break it down to 5% a time. But that's a scam. It would be easier for family budgets to adjust to a gradual increase… They knew a smoker would resist for two or three days and then cave in. But they [were] afraid of a significant jump, which would mobilise the smokers to quit smoking.” (Anonymous, ex-MP).

“[T]he Czechs know that our fiscal situation is such that taxes will simply go up. The trick is not to do this in jumps but via gradual provisions because the market will get used to it.” (Economist and consultant to Czech political parties)

**Table 3:** The tobacco companies' agreed tax harmonization goals and arguments for Central European countries (Czech Republic, Poland, Hungary, Slovakia, Romania).

Goals	Arguments
Oppose any further large-scale increase in total tax incidence [118],[119]	• Large price increases driven by a rapid tax increase would heighten the risk of smuggling. [120]• Smuggling could reduce government revenue from tax; therefore no guarantee that a revenue increase would result from EU harmonisation. [120]• A review of the minimum excise tax level by the European Commission is due in 1996, thus the target level of 57% may be reduced by 2000. [120]
Oppose the requirement of EU's 57% minimum excise level [118],[119]	• Avoid promoting, endorsing or even mentioning this requirement when lobbying governments [118],[119]
Push for derogation in the implementation of the EU minimum excise incidence of 57%, for at least 5 years after integration to the EU [114],[119]	• The EU White Paper on Central and Eastern Europe encourages gradual adoption of EU legislations and preservation of macro-economic stability during the EU accession process. [120]• Rapid restructuring of the tax systems would result in unsustainable price increases, which could seriously damage the economies of the accession countries. [120]• The EU permits acceding countries to request derogations. [120]

PM commissioned Arthur D. Little International (a consultancy company TTCs often used [125]) to conduct an economic impact study to counter proposals for excise increases in the Czech Republic [126]. The study, published in 2000, claimed that the Czech government gained six billion Czech Koruna (approximately US $150 million) from high smoking rates in 1999, due to the reduced healthcare and social costs caused by the early deaths of smokers [126],[127]. This study was subsequently criticised from both economic [128],[129] and moral perspectives [130],[131], and PM was forced to make a public apology [130],[132],[133].

#### Disagreements on implementing the mixed tax structure

Although the Central Europe Tax Task Force acknowledged that a mixed system was required [118], in line with the differences in brand portfolios outlined above, the TTCs had different views as to what mix was preferable [117],[119]. Despite agreeing to push for derogation on the EU's required minimum level of taxation, the industry could not agree on how quickly harmonisation of tax structures should occur in pre-accession, Central European countries [113],[118]. PM wanted to maintain a fully specific system for as long as possible in the Czech Republic and planned to lobby separately on this [134]. BAT, whose focus on cheaper brands [135] would benefit from a system incorporating an ad valorem element (Table 1), preferred faster harmonisation with the EU mixed tax structure [113]. This rift motivated BAT and PM to pursue separate avenues of excise policy influence in the Czech Republic, while also continuing to meet as an industry group.

In 1996, having confirmed “the legal allowance of political contribution” [136], PM organised a “contribution” [137] totalling $300,000 to three Czech political parties that it felt were “consistently pro-free trade and pro-market economy” [138] and “pursued reasonable politics on excise taxation” [138]. These were the three dominant political parties at that time: the Civic Democratic Party (then the senior Government coalition party, led by Vaclav Klaus, then Prime Minister), the Christian and Democratic Union - Czechoslovak People's Party (the second largest party in the Government coalition), and the Civic Democratic Alliance Party (a junior coalition party which held the posts of Minister of Trade and Industry, Minister of Privatisation, and Head of the National Property Fund) [138]. As highlighted by one interviewee:

“They were very clever about it because they made contributions to all [important] parties so that all of them would be in their debt.” (Anonymous, ex-MP)

According to an investigation widely quoted in the Czech media, PM (alongside two steel companies) deliberately sought to obscure the origin of these donations by channelling them through an offshore account [139],[140] (e.g. see footnote 13, p289 in reference 140), a claim which resulted in the resignation of the Deputy Prime Minister, Jiri Skalicky [141],[142],[143]. Two interviewees suggested that transparency in donations to political parties and individual MPs is an ongoing concern.

“Today, unfortunately, the political parties no longer make their sponsors' names public… It's most probably continuing, but in a way we know nothing about.” (Public Health Advocate)

“I remember them [TTCs] paying for something for the MPs, but I can't remember the details now, and we stand no chance of ever finding out. These are experienced people, and they know how to do their business.” (Ex-MP)

BAT appears to have focused its lobbying at the EU level, convening a board-level lobbying visit to the European Commission in November 1997, where they held a series of meetings with high-level European Commission officials, including officials in the Directorate-General for Internal Market and Services and the Directorate-General for Customs and Indirect Taxation, Irish Commissioner Padraig Flynn, United Kingdom Permanent Representative staff and the Vice-President of the European Commission, Sir Leon Brittan [144],[145],[146],[147],[148],[149],[150],[151],[152],[153],[154],[155],[156],[157]. These meetings were designed to facilitate useful, long-term EU connections and signal BAT's importance in Europe [144]. BAT also planned to claim that their business in Eastern Europe suffered from “arbitrary policy-making in key areas, particularly taxation” [144] and that BAT therefore supported “early enlargement” [144] of the EU to expedite adoption of the EU's mixed tobacco excise structure [144]. BAT further planned to offer its “world-wide expertise” [144] on taxation issues to officials in the accession countries and to present itself as a “neutral partner” [144] of the EU and its member states. Our interview data suggest that TTCs are continuing to position themselves as experts on tobacco taxation who can “educate” less-knowledgeable officials:

“You must generate long-lasting relations and you must offer them some specific knowledge. Tobacco tax is a very complex business. There are 200 people in the [Czech] House of Representatives who have all kinds of professions (one is a doctor, another an engine driver, another an engineer). How many of those understand consumer tax on tobacco?” (Anonymous, tobacco industry employee)

In 1998, BAT succeeded in persuading other TTCs to agree on the “BAT path of thinking regarding tax in Central Europe” [158], despite PM's disdain for a mixed structure. Minutes of a BAT-led Central Europe Tax Task Force meeting in January 1998 state PM, Reemtsma, RJR, and BAT unanimously agreed to cooperate on supporting the implementation of a mixed tax structure which complied with EU requirements [159].

#### Outcomes of TTC influence on excise during accession

The Czech Republic was granted two derogation periods on tobacco excise levels when it officially joined the EU in May 2004: 32 months to raise the minimum level to 57% and 44 months to increase the minimum level to 64 euro/1000 cigarettes in the Most Popular Priced Category. This represented a partial success for TTCs, as they had hoped to achieve a five-year derogation period. No derogation was granted on implementing the mixed *structure* [160], which was consistent with BAT's preference, and as of 01 August 2001, a mixed structure (i.e. with both ad valorem and specific components; see Table 2) was introduced.

**Figure 2:**
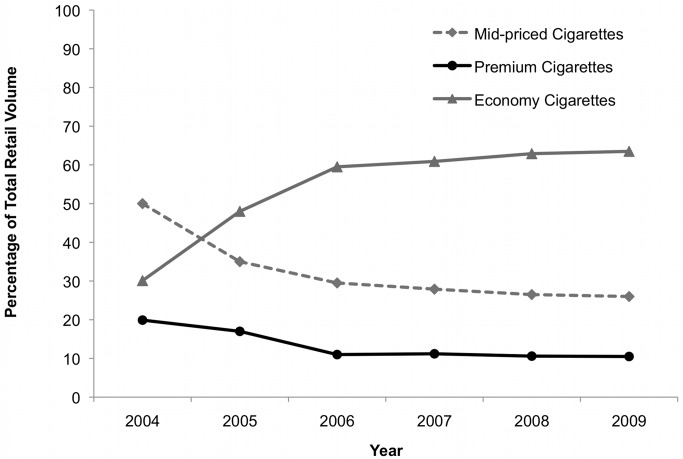
Volume market share by price segment in the Czech Republic, 2004–2009. [174]

The TTCs' shares of the tobacco market in the Czech Republic have changed dramatically since the 2001 change from a specific to a mixed tobacco excise regime. PM's market share dropped from around 80% in 2000 to under 50% in 2010, while BAT's share more than doubled to 21% (Figure 1) [59],[161],[162],[163],[164],[165],[166],[167],[168],[169],[170],[171]. While these changes are consistent with the mixed excise structure advantaging BAT over PM, other factors may have also played a role. First, EU accession in 2004 enabled TTCs to supply cigarettes from any production base in the EU without incurring import duties, effectively removing most of the benefits previously associated with local production [59],[172]. This appears to have prompted BAT and PM to close domestic production facilities in the Czech Republic in July 2004 and 2005, respectively [59],[173]; BAT is now supplying the Czech market solely by imports, while PM retains production at its Kutna Hora factory [59]. Second, in line with trends elsewhere in Europe, an economy cigarette sector quickly emerged around this time [59] and has continued to grow (Figure 2) [174]. This could also have hurt PM's market share as its strategy focused on pushing premium brands, although some analysts suggest PM's failure to improve sales may be attributable to its altered distribution system [59].

**Figure 3:**
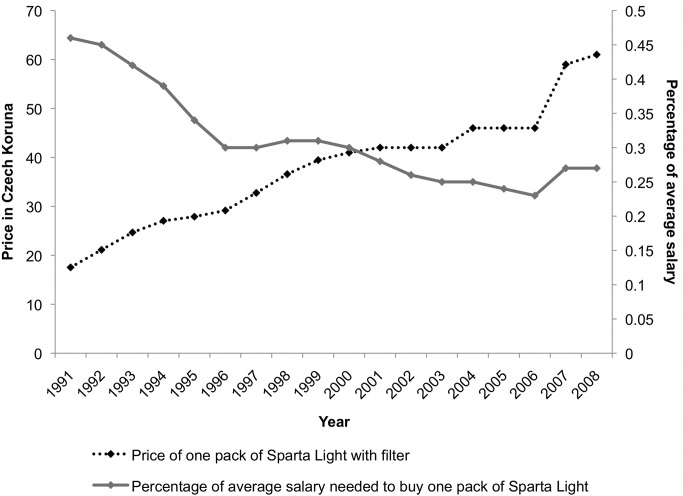
Price of, and the percentage of average salary needed to buy, one pack of cigarettes in the Czech Republic, 1991–2008. [97]

Despite five relatively small excise tax increases between EU accession in 2004 and 2010 [59],[171], cigarettes are becoming increasingly more affordable due to rising income levels (Figure 3). TTCs are taking advantage of this situation by overshifting tax increases and thus increasing profits [174]. Nevertheless, cigarette prices remain low [8] and the country's current tobacco excise yield is one of the lowest in the EU [170].

Given the ineffectiveness of tobacco excise policy in the Czech Republic, it is unsurprising that smoking prevalence rates have changed little since 2000 [175],[176] and that cigarette sales increased between 2000 and 2007 (Figure 1). Although they have since fallen, this decline has been largely attributed to market conditions rather than tobacco control policies [171],[174].

### Continued TTC Influence in the Czech Republic: Interview Data

Since joining the EU, the TTCs have continued their intensive lobbying efforts in the Czech Republic (various interviews), with PM viewed as the most active (interview, civil servant). Political support for the industry appears to be higher than in many European countries with, for example, one of our interviewees arguing that s/he “didn't see a problem with” President Klaus' decision to open the PM's new factory (Czech Member of the European Parliament).

Our interviews provide examples of how PM and the other TTCs continue to court high-level politicians, at both the national and the EU levels, sometimes disguising their involvement:

“I was once invited [last year] to a round-table conference to which legislation experts, customs officers, and the Ministry were invited, and there were representatives from tobacco companies too. It was under the auspices of some training company, but in my opinion, it was the tobacco people who organised it.” (Anonymous, civil servant)

“Last week, there was a social event in Malostranská Beseda [177] organised by Phillip Morris, in which 54 MPs and Senators participated… …We never had 54 come to our [tobacco control] seminars. One or two, maybe…There are 200 MPs and 81 Senators, so 54 is a good amount.” (Public health advocate)

Other interviewees mentioned this expensive reception, one noting that at least one minister attended. Furthermore, one interviewee named three high-level politicians and two members of the presidential team who “represent the views of the industry” (interview, public health advocate), suggesting that industry efforts to court political support remain successful. Interestingly, the tobacco industry interviewee was very keen for us to interview one of these politicians, Senator Kubera, who regularly campaigns against Czech tobacco control proposals [178],[179],[180],[181], claiming:

“Senator Kubera knows about smoking issues about 15 times more than does Dr. Šťastný [an MP currently campaigning for an earmarked excise tax on cigarettes to fund health care]. In any case, his view of the issues is much more relevant than that of Šťastný.” (Tobacco industry employee)

## Discussion

This paper documents extensive evidence of tobacco industry policy influence in the Czech Republic, including over the privatisation process, tobacco advertising, tax levels, and structure. This detailed case study is important as it elucidates industry influence on tax policies, which have not been well researched outside North America [16]. The findings are likely to be particularly relevant for other Eastern Europe countries, many of which experienced similar economic reforms and a process of tobacco industry privatisation around the same time as Czechoslovakia/the Czech Republic. More broadly, the findings draw attention to a range of strategies for influencing policy and gaining market share that tobacco companies may employ in emerging markets. Both privatisation and EU accession provided opportunities for TTC influence. PM was most successful in exploiting the former and BAT the latter, and the successes are reflected in subsequent market share trends (Figures 1 and 2). It is also clear that the industry continues to enjoy high-level political support and access, to a degree that is now rare in many other parts of Europe [182],[183].

On privatisation, we demonstrate that PM attempted to avoid a competitive tender and, having effectively established a production monopoly, sought to influence the monopoly legislation to preclude competition. This substantiates previous evidence that TTCs may attempt to establish monopolies [5], whilst simultaneously extolling the benefits of free market competition [64]. This conduct was previously documented only for BAT [10],[105],[184], but this paper provides evidence of PM acting in the same way.

On advertising, we show that TTCs worked hard to prevent and undermine advertising legislation between 1989 and 1995, ignoring initial legislation and supplanting further proposals for binding advertising bans with a voluntary approach. This is a tactic TTCs have used elsewhere [5],[185],[186],[187] and reveals a consistent industry preference for voluntary over binding controls on marketing [186].

In relation to tobacco excise policy, we document a number of important findings with key relevance for policy. First, in relation to tax levels, both PM and BAT generally aimed for low excise levels. This was particularly so at the point of market entry, when PM was even willing to absorb taxes in order to ensure its brands remained affordable (i.e. to undershift tobacco taxes)—a TTC tactic documented elsewhere [188]. On EU accession, it is clear the industry made concerted and successful efforts to delay the excise increases required. Indeed cigarettes became more affordable post-accession (Figure 3). This indicates that opportunities to improve tobacco control were missed during the accession process, a point that has been previously made [6]. Second, the TTCs angled for gradual, small tax increases (as opposed to intermittent, large increases). This practice is documented during both privatisation and accession and is noted by at least one interviewee. Our data suggest this is because intermittent, large increases are more likely to prompt smokers to quit: a finding consistent with reports of very substantial declines in cigarette consumption following large tax increases in France, Germany and Ukraine [189],[190]. This indicates that intermittent, large tax increases would be more effective as a public health strategy—an issue that requires further research. Third, data show that the industry is currently overshifting tax increases (i.e. increasing cigarette prices, and thus profits, on top of the excise increase) in the Czech Republic. This represents extra profits for TTCs and a lost opportunity for the government, which could have collected this additional revenue as tax. Moreover, the fact that TTCs are overshifting taxes goes against their advice to the government to keep prices low.

In relation to excise structures, our findings support existing studies [16] in suggesting that each TTC had a standard approach to excise structure, with PM promoting a specific structure, designed to narrow the price gap between Marlboro and cheaper brands, and BAT promoting a mixed system, to aid its cheaper brands and disadvantage PM.

To achieve policy influence, TTCs targeted key government officials at both national and EU levels, as they have done elsewhere [10],[105],[191],[192], sometimes exploiting a lack of political and policy expertise in tobacco excise as an opportunity to “educate” politicians. Our interviewees suggest this tactic continues and extends to high-level politicians with whom the industry appears to enjoy significant contact and influence. Political donations to “friendly” political parties were used behind cover, with transparency of political funding identified as an ongoing concern. Other tactics include trying to ensure favourable media coverage and commissioning third-party research to boost credibility of the industry claims; again, tactics noted elsewhere [105],[193],[194].

It is worth noting that for some issues, TTCs' approach and argumentation appear to be context-specific (although always with the ultimate aim of securing corporate advantage, including over competitors). For example, PM supported a tax stamp system in the Czech Republic, once it had secured a dominant position, just as BAT did in Uzbekistan [10]. Yet, elsewhere, PM has lobbied against such a system [105]. This differing stance is explained by the fact that tax stamps protect the interest of dominant TTCs with a domestic base by making it more difficult for their competitors to import tobacco products (including illegally). Market research reports suggest the tax stamp system, and later a ban on selling tobacco from street markets, were effective in limiting the illicit tobacco trade [59].

Empirical evidence suggests several of the arguments developed by TTCs to influence excise policy were misleading. For example, in seeking to delay the implementation of the EU's minimum excise requirement, TTCs planned to argue that raising taxes would increase smuggling. Yet, in reality, the evidence indicates that smuggling is more pervasive in countries with low tobacco tax and loose border regulation [195],[196]. TTCs also agreed to contend that increased tobacco taxes could reduce government revenue, when evidence indicates that tobacco tax increases almost always increase government revenue [197]. Furthermore, the TTCs aimed to exploit the argument that the implementation of tax increases to meet the EU's 57% minimum excise level must be gradual in order to preserve the country's macro-economic stability. In reality, it is unlikely that changes in taxation of tobacco, which is not an essential good [14], would have a significant impact on a country's overall economy [190]. The TTCs' desire for gradual tax increases is more likely to relate to their awareness (as described above) that gradual increases are more easily absorbed by consumers. The fact that a significant derogation period was granted to the Czech Republic in relation to the EU minimum excise requirement (albeit a shorter period than the TTCs were hoping for) suggests the TTCs were relatively successful in influencing this process, despite the flawed nature of their arguments. The chief negotiator of the Czech Republic's accession to the EU was Pavel Telička, a former Member of the European Parliament who now works as a lobbyist in Brussels for large companies [198] and as BAT's EU Social Reporting Facilitator [193].

An important limitation of this study is that the document analysis was based primarily on PM and BAT documents, for the reasons explained in the Methods section. We also found very little information regarding the role played by civil society groups in the development of tobacco control policies in the Czech Republic. We found no industry documents on non-governmental organizations' activities in relation to tobacco advertising or taxation. One document even suggests that industry perceived tobacco control activity to be almost nonexistent in the immediate post-communist period [199]. Although the literature suggests that tobacco-control activities had increased by the late 1990s [200],[201], policy advocacy efforts are reported as limited [202], reflecting the “lack of tradition in civic participation” [202].

Overall, our findings suggest that tobacco industry influence plays a key part in explaining the weak tobacco control policies of the Czech Republic. Improvements in tobacco control will probably be possible only if efforts are made to protect policies from the vested interests of the tobacco industry, as enshrined in Article 5.3 of the FCTC [203], and if public and political attitudes to the industry shift. Transparency in political funding and greater policy advocacy by civil society groups could be important steps towards achieving such a shift. More specifically, our findings point to a number of policy recommendations, particularly in relation to tobacco excise policy (Box 1).

**Box 1. Conclusions and Policy Recommendations**

Recommendations are denoted “*R*” below.

**Tax**

- The tobacco industry will both under- and overshift taxes; its tactics depend on the market structure and economic context. Undershifting is most likely when the market is immature and the tax increase is relatively small. *R: Undershifting can be prevented by substantially increasing tobacco excise taxes.*
- Data suggest the industry is currently overshifting taxes in the Czech Republic. While this is less of an issue for public health, it highlights a missed opportunity for the government to increase tobacco excise. *R: The Czech Republic should consider increasing tobacco excise, particularly specific excise, for the reasons given above.*
- Tobacco industry claims about tobacco excise policy must be treated with extreme caution. Most arguments aim to serve corporate interests and may be inconsistent with established evidence. The complexity of tobacco excise tax policy, perhaps more than any other area of tobacco control policy, enables the tobacco industry to make misleading arguments and to influence policy inappropriately. *R: There is a need to increase understanding among politicians, civil servants, and the public health community of effective tobacco tax policy and of industry efforts to mislead and undermine policy.*
- Large tax increases may benefit public health to a greater extent than incremental increases. *R: Further research is needed to explore this issue.*
- Accession to the EU could provide an opportunity to improve public health, but this paper highlights that it can also provide an opportunity for TTC influence. *R: If and when other countries join the EU, care must be taken to provide appropriate, independent advice on tobacco excise tax policy. Any estimation of the impact of tobacco excise tax increases that is likely to occur with accession needs to allow for likely changes in income (and thus affordability) of tobacco.*
- Both tobacco stamp systems and preventing sales of tobacco from open-air markets may help reduce illicit tobacco trade. *R: These could be effective interventions in other jurisdictions.*

**TTC Political Links**

- Tobacco-control policies and therefore the health of the public suffer when policymakers maintain connections with the TTCs, as this provides the TTCs a direct avenue for policy influence. *R: Article 5.3 of FCTC, if properly implemented, can address this but requires industry actions to be monitored and exposed and greater public and political awareness of industry tactics.*

**Advertising**

TTCs will work hard (often collectively) to prevent any significant restrictions on their ability to promote their products. Legislation may be ignored by TTCs if it is not sufficiently clear, and TTCs are likely to propose voluntary advertising restrictions in order to avoid binding legislative. *R: Legislation must be watertight; voluntary advertising restrictions are inadequate.*

**Privatisation**

There is now substantial evidence that, in the context of privatisation, TTCs will: (i) attempt to establish monopoly positions while harnessing the benefits of market liberalisation to access new markets; (ii) seek to keep cigarette excise rates low; and (iii) ensure the freedom to promote tobacco products. *R: Tobacco control measures including a comprehensive advertising ban should be implemented and enforced prior to privatisation.*

## Supporting Information

Alternative Language Abstract S1 Japanese translation of the abstract by RS.
(DOCX)

Alternative Language Abstract S2 Czech translation of the abstract by HR and Eva Kralikova.
(DOC)

## Abbreviations

BAT: British American Tobacco
EU: European Union
FCTC: Framework Convention on Tobacco Control
MP(s): Member(s) of Parliament
PM: Philip Morris
RJR: R.J. Reynolds
Tabak: Tabak Akciová Společnost (a.s.)
TTC(s): transnational tobacco company(ies)
