# Supplementary material for: Tobacco Industry Manipulation of Tobacco Excise and Tobacco Advertising Policies in the Czech Republic: An Analysis of Tobacco Industry Documents
Source: PLoS Med. 2012 Jun 26;9(6):e1001248. doi: 10.1371/journal.pmed.1001248 (PMC3383744; doi:10.1371/journal.pmed.1001248)
Supplement: Alternative Language Abstract S1 — Japanese translation of the abstract by RS. (DOCX) [file pmed.1001248.s001.docx]

**チェコ共和国におけるたばこ消費税・たばこ広告政策に対するたばこ企業の操作：企業内部書類の解析**

**抄録**

**背景：**ヨーロッパの中でチェコ共和国のたばこ対策における経歴は特に乏しい。この論文では、多国籍たばこ会社（以下、TCCs）がどのようにチェコ共和国のたばこ政策に影響を及ぼしてきたかを検証する。この論文でたばこ税政策に焦点を当てているのは、高い課税率がたばこ消費量を効果的に減らす手段の一つであると共に、税構造がタバコ会社の競合性を駆り立てる重要な一因であるからである。

**手法と結果：**1989年から2004/5年付けのたばこ企業内部書類をLegacy Tobacco Documents Library のホームページにて検索後、socio-historical approach を用いて解析し、情報提供者のインタビューや２次データと照合した。TTCs内部書類は、たばこ企業がたばこ対策に多大な影響を及ぼしていた事を明らかにしている。具体的に、フィリップ・モリス社は、たばこ宣伝活動に対する制限を無視、転覆、又は弱体化し、拘束力のある法律ではなく、自粛を基本とするたばこ対策の推進に努めた。フィリップ・モリス社とブリティッシュ・アメリカン・タバコ社はそれぞれ、自社に有利なたばこ税構造の構築のためにロビー活動を行い、順番に成功を収めた。さらに、TTCsは、たばこ税率を低く保ち、大幅な税率の引き上げを防ぐために協調して活動を行った。様々な論理を展開して行ったロビー活動により、たばこ企業はヨーロッパ連合に加盟する際に必要とされるチェコ共和国のたばこ税率引き上げの施行を遅らせる事に成功した。チェコ共和国内では、ヨーロッパ連合加盟後、タバコの価格の水準は購入し易いものになった。また、TTCsは低いたばこ税を利用する形で価格の上乗せを行って利益を得ていた。情報提供者へのインタビュー結果は、現在もTTCsが上級の政治家のサポートを得て、政策に影響を及ぼすための活動を積極的に続けている事を示唆している。

**結論：**多国籍たばこ会社がチェコ共和国において、たばこの宣伝・課税に関する政策に影響を及ぼしていたこと、この影響は現在も続いていることは明らかである。そして、これらはチェコ共和国の緩いたばこ対策の記録を説明すると結論付けられる。チェコ共和国ではたばこ税を引き上げる余地がかなりあり、短期間で大幅に増税することが喫煙率の減少に最も効果的である。
