# Supplementary material for: Tobacco Industry Manipulation of Tobacco Excise and Tobacco Advertising Policies in the Czech Republic: An Analysis of Tobacco Industry Documents
Source: PLoS Med. 2012 Jun 26;9(6):e1001248. doi: 10.1371/journal.pmed.1001248 (PMC3383744; doi:10.1371/journal.pmed.1001248)
Supplement: Alternative Language Abstract S2 — Czech translation of the abstract by HR and Eva Kralikova. (DOC) [file pmed.1001248.s002.doc]

TITUL:

**Manipulace tabákového průmyslu ČR při ovlivňování výše spotřebních daní a regulace reklamy: analýza dokumentů tabákového průmyslu**

AUTOŘI:

Risako Shirane (1), Katherine Smith (2), Hana Ross (3), Karin E Silver (4), Simon Williams (5), Anna Gilmore(6)*

*= korespondující autor

**ABSTRAKT**

**Úvod:** Kontrola tabáku je v České republice jedna z nejslabších v Evropě. Tato práce zkoumá snahy nadnárodních tabákových společností (NTS) ovlivnit legislativu se zvláštním zřetelem na spotřební daně, protože právě vysoké daně jsou účinnou prevencí užívání tabáku a jejich struktura je důležitým aspektem konkurenceschopnosti NTS.

**Metody a výsledky:**. Dokumenty nadnárodních tabákových společností z let 1989–2004/5 získané z webové stránky Legacy Tobacco Documents Library byly po sociálně-historické analyze porovnány s informacemi klíčových osobností a sekundárními daty. Dokumenty prokazují klíčovou roli průmyslu na přístup ke kontrole tabáku. Philip Morris (PM) ignoroval, překroutil a oslabil různé pokusy o omezení reklamy na tabák při prosazování dobrovolné regulace jako alternativy k závazným právním předpisům. PM a British American Tobacco (BAT) se *strukturu* tabákových daní se snažili ovlivnit odděleně - každý se snažil prosadit takovou úpravu, která zvýhodňuje vlastní portfolio značek před konkurencí úspěch ze svého pohledu. Pokud jde o *výši* spotřebních daní, jednotliví výrobci více spolupracovali, aby udrželi jejich nízkou úroveň a zabránili jejich výraznému zvyšování. Společné lobování za použití řady zavádějících argumentů, bylo úspěšné a oddálilo zvýšení daní podle požadavků Evropské unie.

Na rozdíl od argumentů průmyslu ukazují data, že cigarety se po vstupu do EU staly *dostupnějšími* a že nízké daně umožnily při zvyšování cen vyšší profit pro NTS. Data z rozhovorů ukazují, že NTS požívají politickou podporu na nejvyšší úrovni a pokračují v aktivním ovlivňování politiky.

**Závěr:** Existují jasné důkazy o minulém i současném vlivu NTS na tabákovou reklamu a spotřební daně a tento vliv pokračuje. To pomáhá objasnit nízkou úroveň kontroly tabáku v této zemi. Z dat vyplývá, že v České republice existuje významný prostor pro zvýšení tabákových daní a že výrazné (spíše než malé, postupné) zvýšení daní je nejúčinnějším opatřením pro snižování prevalence kouření.
